# Heart Rate Variability and Cardiorespiratory Fitness in Youth Populations: The Impact of Heart Rate on Data Analysis

**DOI:** 10.5114/jhk/159581

**Published:** 2023-01-20

**Authors:** Abel Plaza-Florido, Jerzy Sacha, Juan M. A. Alcantara

**Affiliations:** 1 PROFITH “PROmoting FITness and Health Through Physical Activity” Research Group, Sport and Health University Research Institute (iMUDS), Department of Physical and Sports Education, Faculty of Sport Sciences, University of Granada, Granada, Spain.; 2 Faculty of Physical Education and Physiotherapy, Opole University of Technology, Opole, Poland.; 3 Department of Cardiology, University Hospital, University of Opole, Opole, Poland.

**Keywords:** parasympathetic activity, autonomic nervous system, R-R interval, vagal activity

## Abstract

The positive association between heart rate variability and cardiorespiratory fitness in youth populations is unclear. In this regard, several methodological aspects related to heart rate variability analysis could partially explain the disagreement between studies. To the best of the authors’ knowledge, the influence of the heart rate on data analysis is unclear. In the present short communication, we discuss the impact of the heart rate on the associations between heart rate variability and cardiorespiratory fitness in youth. In addition, we proposed some aspects that should be considered for statistical analyses when the relationship between heart rate variability and cardiorespiratory fitness is studied. Finally, we should acknowledge that these recommendations may be applicable for other health-related outcomes different than cardiorespiratory fitness (e.g., inflammatory markers, cognition, cardiovascular disease status).

## Short Communication

Cardiac autonomic modulation can be determined using non-invasive biomarkers such as heart rate variability (HRV), i.e., variations on the time between consecutive heart beats or the time between R-R peaks (Task Force, 1996). HRV can be expressed using different variables (Table 1) which are derived from the measured R-R signal (henceforth *HRV variables*) (Task Force, 1996). Usually, low values of HRV variables under resting conditions (low parasympathetic modulation) have been related to a higher risk of cardiovascular disease (CVD) and mortality (Task Force, 1996) as well as poor athletic performance (Chiu et al., 2022; Pagaduan et al., 2020). Furthermore, youth's low cardiorespiratory fitness (CRF) levels are related to a higher risk of CVD and mortality later in life (Högström et al., 2014). Interestingly, a logistic regression model using resting HRV variables can identify children with low CRF levels (estimating their CRF) without performing any specific exercise test (Redón et al., 2020). This could be practical for clinicians and exercise physiologists as allows a fast detection of youths with a higher risk of developing CVD later in life. However, the literature showing a positive association between HRV variables and CRF levels in youth is inconsistent (Oliveira et al., 2017; Plaza-Florido et al., 2021a). In this regard, diverse methodological aspects (e.g., length of the R-R signal recording, artefact correction and data analysis) could partially explain the disagreement between studies (Plaza-Florido et al., 2021a).

**Table 1:** Common heart rate variability (HRV) variables which are considered indicators of parasympathetic modulation during resting recordings.

HRV variable abbreviation	Full name
**Time-domain**	
RMSSD (ms)	The square root of the mean squared differences of successive NN intervals
SDNN (ms)	The standard deviation of the NN interval
pNN50 (%)	The percentage of pairs of adjacent NN intervals differing by more than 50 ms in the entire recording
**Frequency-domain** TP (absolute units; ms^2^) HF (absolute units; ms^2^) LF (absolute units; ms^2^)	Total power of HRV spectrum The absolute power of the High Frequency band (HF: 0.15–0.4 Hertz) The absolute power of the Low Frequency band (HF: 0.04–0.15 Hertz)

TP: total power; HF: high frequency; LF: low frequency; ms: milliseconds; NN intervals: normal R-R intervals in an electrocardiogram. Abbreviations and full name of HRV parameters are extracted from Task Force Report (1996).

**Figure 1:**
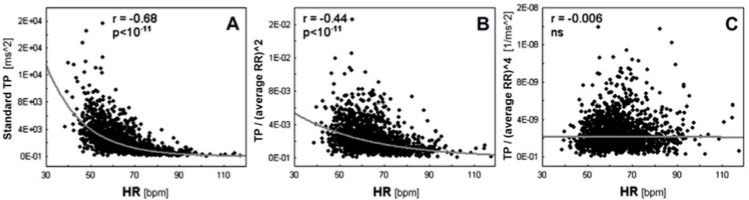
Associations between HR and uncorrected (Panel A), partially corrected (Panel B) and fully corrected (Panel C) heart rate variability (HRV). *Panel A shows the inverse association between HRV, i.e., total power (TP) of HRV spectrum and HR, while Panel B and C show the associations after dividing TP by the average R-R interval (RR) to the power 2 and the power 4, respectively. The examples show that in order to fully correct HRV for the prevailing HR, one must select the appropriate power of the average R-R interval—in this case, TP completely loses dependence on the HR only after dividing by the average R-R interval to the power of 4. Reprinted with modification from Sacha et al. (2013)*.

To our knowledge, the less explored methodological aspect may be the influence of the heart rate (HR) on data analysis (Sacha, 2014a). It is well-known that an inverse and non-linear association exists between HRV variables and the HR (Sacha, 2014a). People exhibiting a low HR usually present higher HRV compared to people exhibiting an elevated HR, an issue which may introduce a mathematical bias in analyses (Sacha, 2014a). Likewise, certain associations showed in previous research (Plaza-Florido et al., 2019a) and CVD risk factors could result from the HR rather than HRV (Sacha, 2014b). Therefore, HRV variables should be “corrected” considering the prevailing HR, i.e., HRV should be calculated relative to the average HR (Sacha, 2014b). To this end, HRV variables should be divided by the appropriate powers of the average R-R interval (Sacha, 2014b). After performing this “correction procedure”, the association between HRV variables and the HR disappears (Figure 1) (Sacha et al., 2013). Including this correction procedure into analyses allows to explore whether the HR influences (over-powers) the “real” clinical value of HRV variables, i.e., thus we are able to determine the HR contribution to the clinical value of HRV in a given clinical context (Sacha, 2014b).

This correction procedure has been used in different studies observing the relationship between HRV variables and CRF levels in youths (Grant et al., 2013; Plaza-Florido et al., 2019a). Grant et al. (2013) reported that pNN50 (Table 1) was positively associated with CRF in healthy young adults (18–22 years old), while the HR was negatively and stronger associated with CRF. Importantly, the corrected HRV variables were not associated with CRF (Grant et al., 2013). Similar results were reported by Plaza-Florido et al. (2019a) in a study addressing the associations between HRV variables and CRF relative to body weight (CRFBW) in children with overweight/obesity (9–11 years old). RMSSD, pNN50, and SDNN (Table 1) were positively associated with CRFBW (Plaza-Florido et al., 2019a). However, these associations were not observed after performing the correction procedure detailed above (Plaza-Florido et al., 2019a). In contrast, the HR showed stronger association with CRF_BW_ compared to HRV variables (Plaza-Florido et al., 2019a). These findings are in agreement with those recently published on a cohort of healthy young adults (20–24 years old) (Plaza-Florido et al., 2021b). Besides, in the abovementioned study on healthy young adults, CRF was objectively assessed using indirect calorimetry and an incremental exercise test, while Grant et al. (2013) estimated CRF using a field test (the Cooper 12- min run test). Nevertheless, all these studies (both performed on healthy young adults (Grant et al., 2013; Plaza-Florido et al., 2021b), and the one on children with overweight/obesity (Plaza-Florido et al., 2019a) agreed and concluded that the HR was better associated with CRF (i.e., CRFBW) than HRV variables.

On the other hand, other studies reported associations between HRV variables and CRF considering the confounding role of the HR (Chen et al., 2019; Leppänen et al., 2019; Michels et al., 2013), although they did not compare whether HRV variables or the HR were better associated with CRF. Leppänen et al. (2019) reported that RMSSD, SDNN, and HF (Table 1) were positively associated with CRF (expressed as Watts per kilogram of lean body mass) in boys (6–9 years old). However, only the corrected RMSSD was positively associated with CRF (Leppänen et al., 2019). Interestingly, Michels et al. (2013) observed, after including the HR as a covariate in multiple regression models, a positive association between RMSSD, pNN50, and HF and CRF estimated using a field test (the 20 m shuttle test) in boys (5–10 years old). Recently, Chen et al. (2019) performed cross-sectional analyses in a large cohort of 2316 healthy middle-aged adults, and they observed that the better was CRF (represented by exercise duration—time to volitional exhaustion), the higher were HRV variable values (SDNN and RMSSD). Unfortunately, the correction procedure was not performed in the latter studies (Chen et al., 2019; Michels et al., 2013), although they included the HR as a covariate in statistical analyses and the associations between HRV and CRF remained significant (Chen et al., 2019; Michels et al., 2013).

In general, associations between HRV variables and CRF could be (at least partially) explained by the “confounding” role of the HR (Grant et al., 2013; Plaza-Florido et al., 2019a, 2021b). This “HR impact” could be of interest for clinicians as the HR presents certain advantages compared to HRV variables; the most evident one is that HR data is much easier to obtain and interpret than HRV variables. Nevertheless, in most of the studies, the associations of HRV variables and the HR with CRF were weak to moderate (r or β statistical coefficients ranged from 0.19 to 0.32 for HRV variables, and from -0.33 to -0.41 for the HR, respectively). The two studies that included the HR as a covariate (Chen et al., 2019; Michels et al., 2013) observed, although this is a usual observation in most of the studies, a positive association between HRV variables and CRF. In line with their findings, one study reported that differences in HRV among children with different weight status might be explained by their differences in the HR regardless the statistical approach (i.e., corrected HRV variables or adding the HR as a covariate) (Plaza-Florido et al., 2019b). In this regard, and as their influence is difficult to be elucidated, we should acknowledge that other methodological aspects regarding the HRV assessment and processing (e.g., length of the R-R signal recording, artefact correction, etc. (Plaza-Florido et al., 2021a)), as well as different methodologies to determine CRF (e.g., estimated from field tests or measured by indirect calorimetry) could also contribute to the discrepancies between studies (i.e., the HR has an impact on the associations between HRV variables and CRF).

Undoubtedly, more research and studies are warranted on this topic. Indeed, further studies investigating the impact of the HR correction procedure on the associations between HRV variables and different health-related outcomes (e.g., adipokines, cognition, etc.) are needed. In our humble opinion, the following aspects should be considered in the analysis approach when associations between HRV variables and health-related outcomes are studied. Firstly, researchers should test these associations as well as the HR relationship using independent statistical models (e.g., HR and HRV variables as predictors/independent variables in different regression models). Then, if both HRV variables and the HR are associated with the health-related outcomes, the correction procedure should be considered (i.e., calculating HRV variables divided by the suitable powers of the average R-R interval) and replicate all the analyses using the corrected HRV variables instead. Alternatively, the HR can be included as a covariate in the multiple regression model when the associations are assessed. However, it is important to highlight that HRV variables and the HR cannot co-exist in the same statistical model as predictors/independent variables due to the strong correlation between them (i.e., multicollinearity). Another option is to add the corrected HRV variables and the HR together as predictors/independent variables in the same statistical model—as the corrected HRV variables are not correlated with the HR after performing the correction procedure (Sacha, 2014a). Considering all these steps in the analysis approach might allow to determine the “real” relationship between HRV variables and diverse health-related outcomes, and to study whether they are better associated with these outcomes than the HR. Lastly, it should be noted that this correction procedure and the analysis approach could also be performed in studies in which the outcome variables are associated with the HR (Sacha, 2014b).
